# Supplementary material for: AoSsk1, a Response Regulator Required for Mycelial Growth and Development, Stress Responses, Trap Formation, and the Secondary Metabolism in Arthrobotrys oligospora
Source: J Fungi (Basel). 2022 Mar 3;8(3):260. doi: 10.3390/jof8030260 (PMC8952730; doi:10.3390/jof8030260)
Supplement: Supplementary file 1 [file jof-08-00260-s001.zip › jof-1611051-supplementary.pdf]

Supplementary materials

Figure S1. Phylogenetic tree analysis based on the amino acid sequences of Ssk1 homologous proteins from different fungi. GenBank accession numbers are provided in brackets. The amino acid sequences of Ric8 proteins were aligned with ClustalX version 1.83, and MEGA 7 was used to construct a neighbor-joining tree, including bootstrap analysis with 1,000 replicates. Numbers below nodes indicate the bootstrap value. The bar marker indicates the genetic distance, which is proportional to the number.

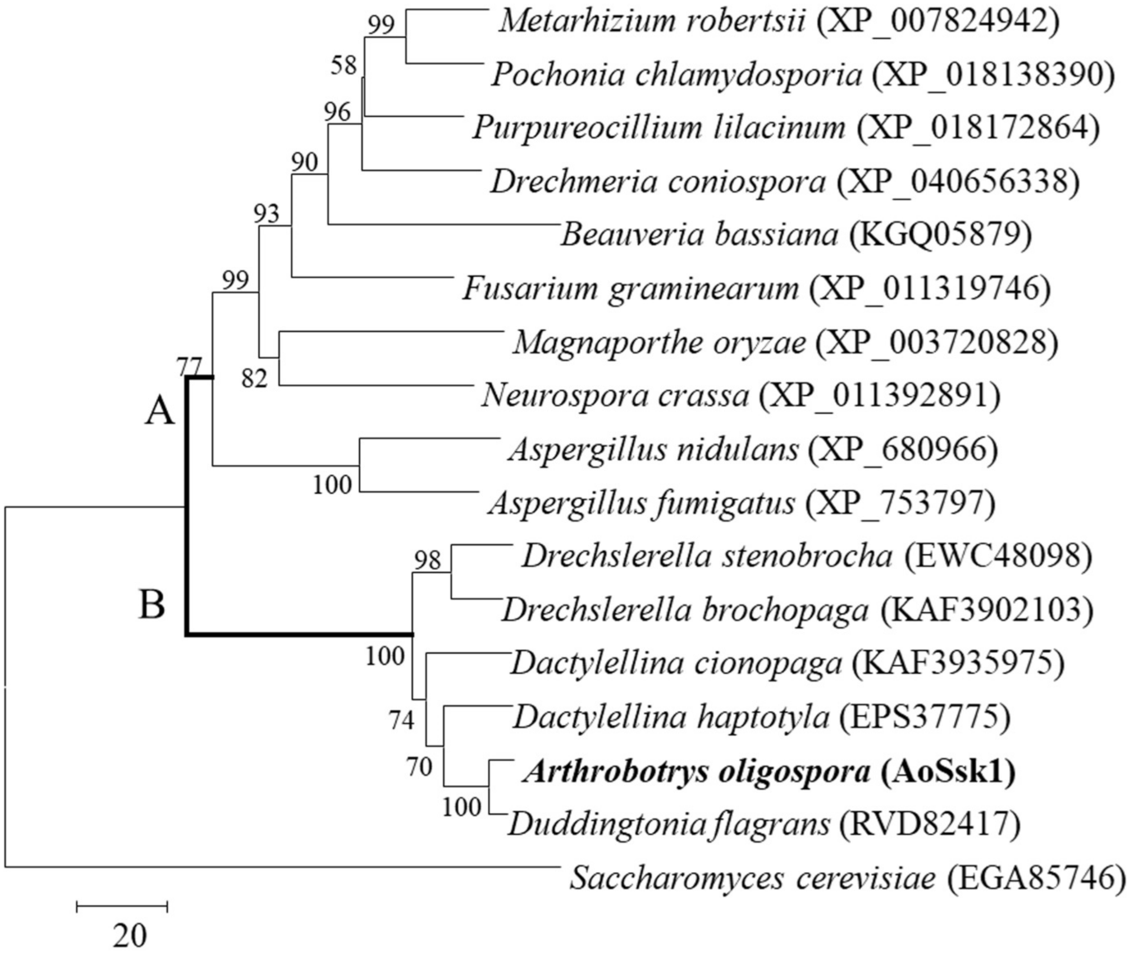

Figure S2. ***Aossk1*** **knock-out and verification in** ***A. oligospora*****.** A. Diagrammatic sketch of homologous recombination. B. The diagrammatic sketch of homologous recombination of *Aossk1* and the homologous flanks of the target gene. C. *Aossk1*-deleted transformants were confirmed by PCR method. D. Southern blotting analysis of wild-type (WT) and transformant.

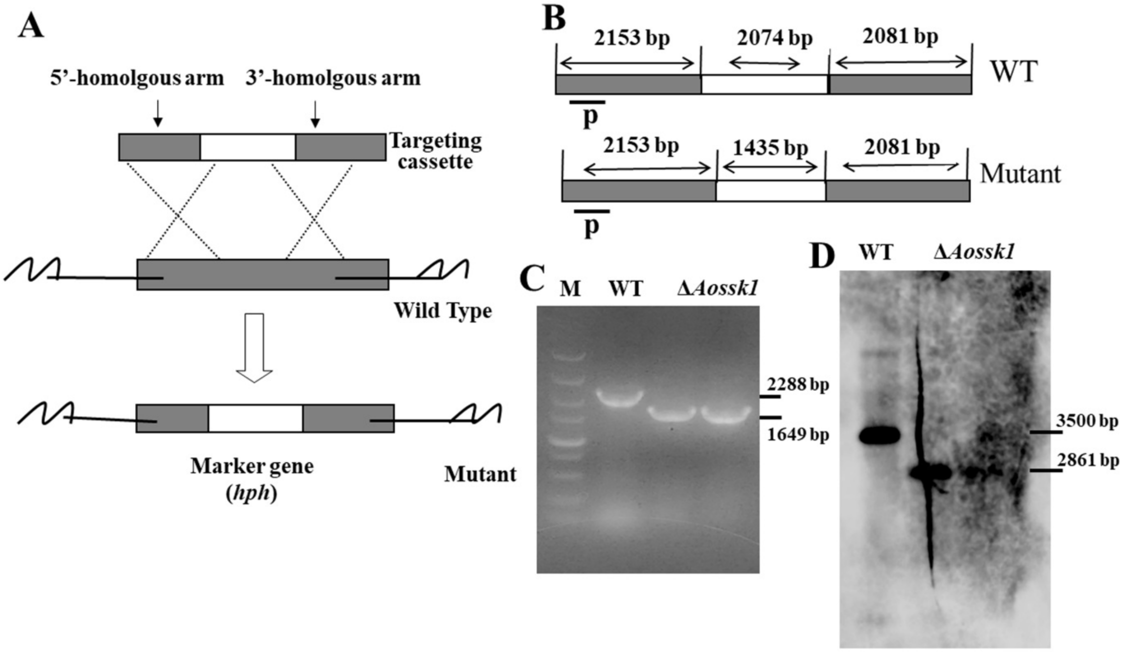

Figure S3. Comparison of hyphal septum and cell nucleus between the WT and mutants. A. The hyphal septum of the WT and mutants were stained with 20 μg/mL calcofluor white (CFW). B. Hyphae of the WT and Δ*Aossk1* mutant strains were stained with CFW and 4',6-diamidino-2-phenylindole (DAPI); samples were examined using an inverted fluorescence microscope. Bar=10 μm.

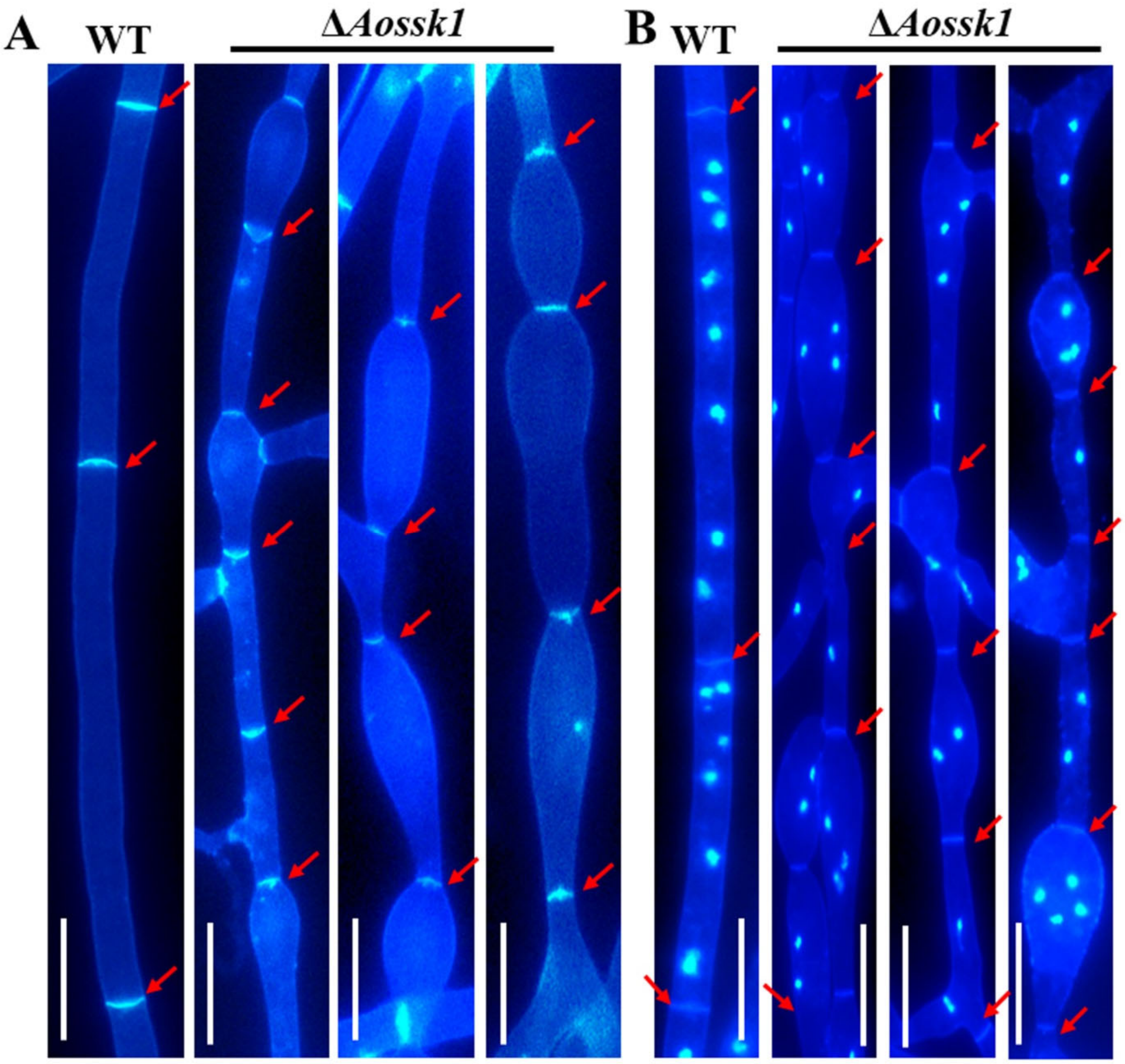

Figure S4. Comparison of oxidative stress response between WT and mutant strains. A. Colonial morphology of fungal strains under oxidative stress. B. Relative growth inhibition (RGI) of fungal colonies after being grown for 6 days at 28°C on TG plates supplemented with different concentration of menadione and H_2O_2. An asterisk indicates a significant difference between Δ*Aossk1* mutant and the WT strain ($p < 0.05$).

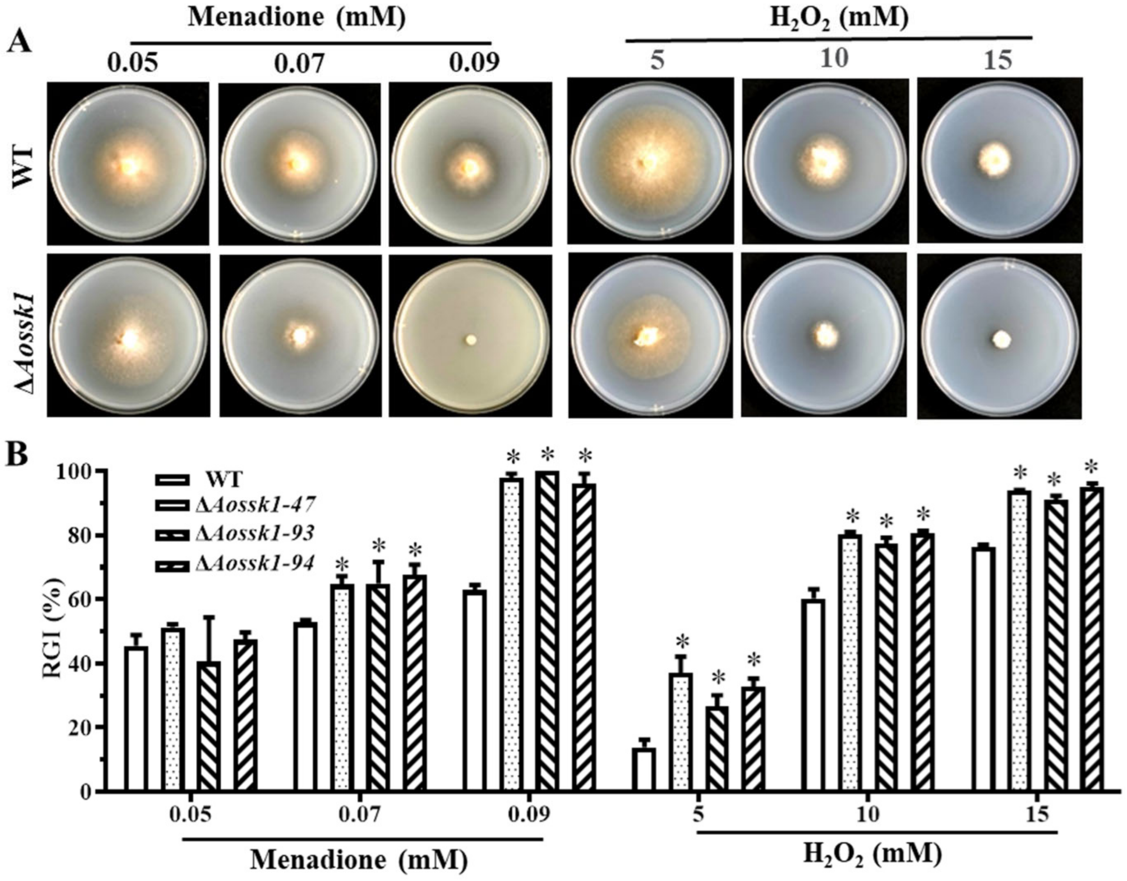

Figure S5. Comparison of cell wall-interfering stress between WT and mutant strains. A. Colonial morphology of fungal strains under cell wall-interfering stress. B. Relative growth inhibition (RGI) of fungal colonies after being grown for 6 days at 28°C on TG plates supplemented with different concentration of Congo red and SDS. An asterisk indicates a significant difference between Δ*Aossk1* mutant and the WT strain ($p < 0.05$).

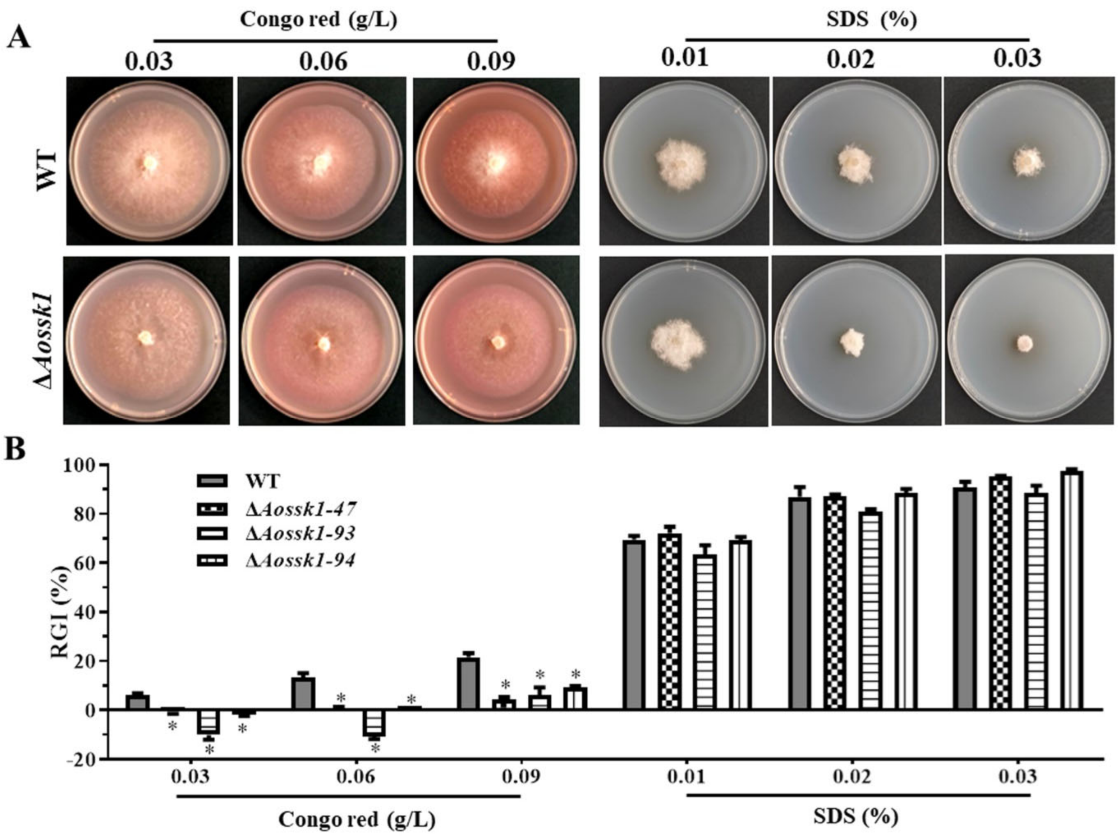

Figure S6. Comparison of ion chromatogram of arthrobotrisin between WT and mutant strains. Arrow, the peak of arthrobotrisin.

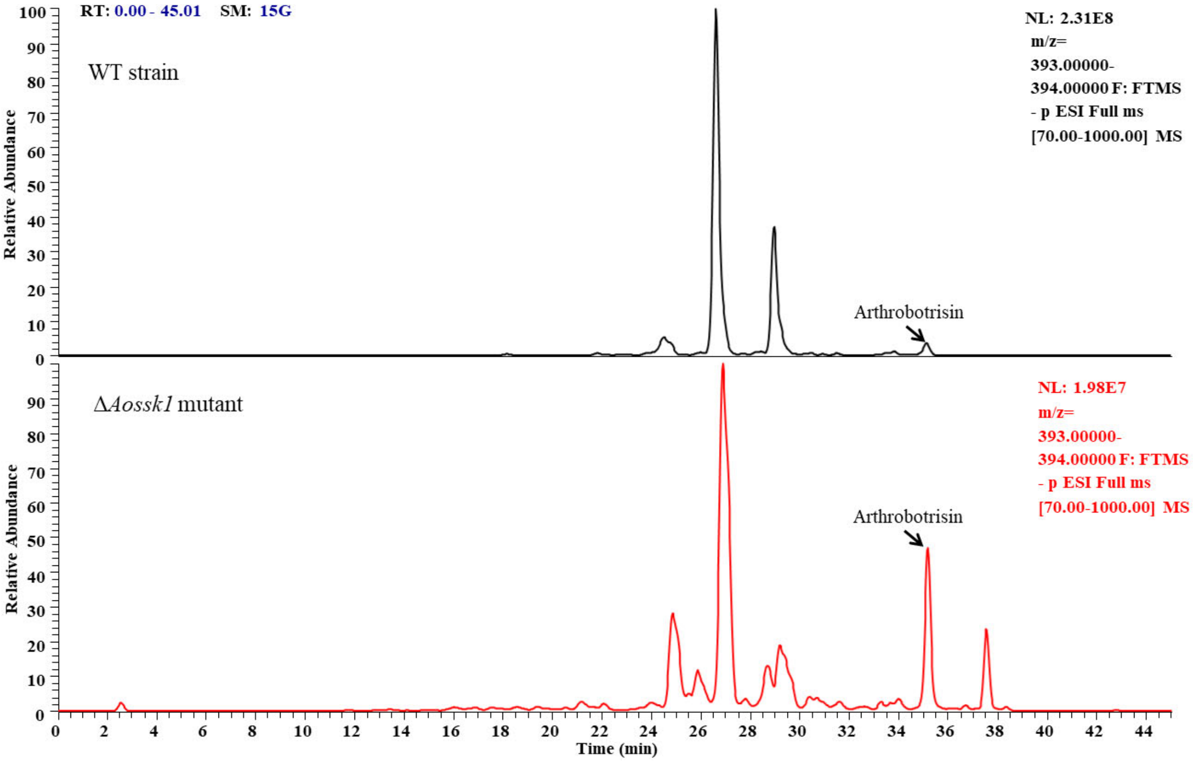

Table S1. The sequence similarity between AoSsk1 and other orthologs from different fungi. The sequence similarity of orthologous **AoSsk1** was analyzed using DNAman software.

Fungal species	Simialrity to AoSsk1 (%)	Groups
Arthrobotrys oligospora	100	NT fungi
Duddingtonia flagrans	90.5	
Drechslerella brochopaga	79.6	
Dactylellina haptotyla	66.9	
Dactylellina cionopaga	62.9	
Drechslerella stenobrocha	57.3	
Neurospora crassa	45.2	Filamentous fungi
Aspergillus nidulans	44.1	
Aspergillus fumigatus	34.3	
Purpureocillium lilacinum	34.1	
Pochonia chlamydosporia	30.0	
Metarhizium robertsii	45.9	
Drechmeria coniospora	32.0	
Beauveria bassiana	46.7	
Fusarium graminearum	36.2	
Magnaporthe oryzae	50.1	
Saccharomyces cerevisiae	22.5	Yeast

Table S2. Primers used for genetic manipulation in this study.

Primers	Sequence (5′-3′)	Description
Aossk1-5f	GTAACGCCAGGGTTTTCCCAGTCACGACGTAGGGATCGGATCGTAGGTG	Amplify the *Aossk1* gene 5' flank
Aossk1-5r	ATCCACTTAACGTTACTGAAATCTCCAACTCTTCGTCTACCCCACAACC	
Aossk1-3f	CTCCTTCAATATCATCTTCTGTCTCCGACGAGATGCCCGACTGCTAGAC	Amplify the *Aossk1* gene 3' flank
Aossk1-3r	GCGGATAACAATTTCACACAGGAAACAGCTTCAGCTCAGACCGAAACCT	
hphF	GTCGGAGACAGAAGATGATATTGAAGGAGC	Amplify the *hph* cassette
hphR	GTTGGAGATTTCAGTAACGTTAAGTGGAT	
yz-5f	TTCATACACCTCGCACAA	Verify the transformants
yz-3r	TTACCGTTTATCTCAGTTCC	
tz-5f	ATAAGGGTAGTGTAGACAAGGA	Make Southern blotting probe
tz-3r	GGAAGAAGACGGAGGAGAAC	

Table S3. List of RT-PCR primers used in this study.

Sporulation-related genes	Sequence (5′-3′)
AOL_s00043g361 (*fluG*)	361-5F-GATTCCAGTCCCGTGAATTC
	361-3R-GCTAAGGAGAGGATGGGCAT
AOL_s00097g514 (*brlA*)	514-5F-TTGAGGCCTCGATCCGTAGA
	514-3R-AGGTAGATGGCGCTGTTACG
AOL_s00173g221 (*wetA*)	211-5F-TTACATGCCACCCCAAGTCC
	211-3R-CAATTGCAACTGCGTCCACA
AOL_s00054g811 (*velB*)	811-5F- ATTCCGCAACTTCTCCCTCA
	811-3R- GGCATGTTTGGATTCTGGGG
AOL_s00080g63 (*abaA*)	63-5F-AACTTTATGCGCCTTGTCGT
	63-3R-TTGGCTAGGTGGTCTGTACG
AOL_s00007g157 (*flbC*)	157-5F-CTCTCCGGCAAAGACAATCG
	157-3R-GTCGACTGAGGATAGTAGCT
AOL_s00006g570 (*rodA*)	570-5F- GCGGATCCAACATGAAGCTT
	570-3R- GGTTGACAACTGGGATGCTG
**Genes related to fatty acid oxidation**	**Sequence (5′-3′)**
AOL_s00004g288	288-5F-AAGAAATCCCACTTCAGAGAGG
	288-3R-TACGTGTCCAGTAACATAGCTC
AOL_s00081g51	51-5F-GCCGATCCTTACCAAATCATTC
	51-3R-CCAATTCTTTTCCGTAGCTGAG
AOL_s00210g122	122-5F-GCCGCACATATTGTTAACAGAT
	122-3R-TGATCTTGCTGTTCTCAGTCAT
AOL_s00110g113	113-5F-CTAACAGAAACTCAAGCATCGG
	113-3R-GGAACCGGATTCATGAAATGAG
AOL_s00079g276	276-5F-AACAATCCGTCGTTATTGTTCC
	276-3R-GCGATCATGTAGTCTAGTCCTC
AOL_s00054g29	29-5F-GGTATCTACGGAAATTTTGGCC
	29-3R-GTGCAATATAATCGGGCTTGAG
AOL_s00004g606	606-5F-TTCGGATTCGTTATTACCTCCC
	606-3R-TAACATGAGTCGCTTGTTTGTG
β-tubulin gene	tub-5F-CCACCTTCGTCGGTAACTC
AOL_s00076g640 (*tub*)	tub-3R-TCGTCCATACCCTCACCAG

Table S4. Comparison of the differential compounds-metabolic pathways between the Δ*Aossk1* mutant and WT strain.

Metabolic pathway	Upregulated compounds		Downregulated compounds	
	Count	Percent (%)	Count	Percent (%)
Superpathway of aromatic compound degradation via 2-oxopent-4-enoate	2	0.24	81	1.11
Anaerobic aromatic compound degradation (Thauera aromatica)	2	0.24	75	1.03
Superpathway of lipoxygenase	10	1.21	50	0.69
Superpathway of trichothecene biosynthesis	4	0.49	55	0.76
Superpathway of chorismate metabolism	1	0.12	53	0.73
Superpathway of aerobic toluene degradation	1	0.12	55	0.76
Superpathway of aromatic compound degradation via 3-oxoadipate	0	0	50	0.69
Superpathway of steroid hormone biosynthesis	3	0.36	41	0.56
Superpathway of gibberellin biosynthesis	3	0.36	43	0.59
Superpathway of scopolin and esculin biosynthesis	0	0	36	0.49
Naphthalene degradation to acetyl-CoA	1	0.12	39	0.54
Toluene degradation III (aerobic) (via p-cresol)	1	0.12	42	0.58
Novobiocin biosynthesis	3	0.36	34	0.47
Superpathway of 4-hydroxybenzoate biosynthesis (yeast)	1	0.12	37	0.51
Superpathway of rosmarinic acid biosynthesis	1	0.12	32	0.44
Toluene degradation IV (aerobic) (via catechol)	0	0	36	0.49
Superpathway of aromatic amino acid biosynthesis	1	0.12	31	0.43
Superpathway of cholesterol degradation I (cholesterol oxidase)	2	0.24	21	0.29
Superpathway of cholesterol degradation II (cholesterol dehydrogenase)	2	0.24	21	0.29
Other	32	3.88	322	4.43
<Not Set>	754	91.5	6120	84.14

Table S5. Top 20 annotated compounds enriched in KEGG analysis.

Compound name	Formula	Molecular Weight	RT [min]	Log2 Fold Change: Δ*Aossk1*/WT	P-value: Δ*Aossk1*/WT
(2,6-Dimethylfuro[2,3-f][1]benzofuran-3,7-diyl)bis[(4-methyl-1-piperazinyl)methanone]	$C_{24}H_{30}N_4O_4$	438.2255	22.926	-4.44	0.01494
1-Deoxy-1-[4-({(1R)-1-[(7S)-4-hydroxy-2-imino-7-methyl-1,2,7,8-tetrahydro-6-pteridinyl]ethyl}amino)phenyl]-D-ribitol	$C_{20}H_{28}N_6O_5$	432.2141	32.414	-4.69	0.00746
ethyl 1-[4-({[4-(tert-butyl)anilino]carbonyl}amino)phenyl]-3,5-dimethyl-1H-pyrazole-4-carboxylate	$C_{25}H_{30}N_4O_3$	434.2298	28.48	-8.73	0.00016
Delcosine	$C_{24}H_{39}NO_7$	453.272	31.51	-2.08	0.01715
2-(1H-Indol-3-ylmethyl)-3-oxo-3-{[2-phenyl-1-(phenylalanylamino)ethyl]amino}propanoic acid	$C_{29}H_{30}N_4O_4$	498.2248	30.749	-4.33	0.00081
Isodomedin	$C_{22}H_3O_6$	392.2196	24.552	-4.43	0.00987
ethyl 1-[4-({[4-(tert-butyl)anilino]carbonyl}amino)phenyl]-3,5-dimethyl-1H-pyrazole-4-carboxylate	$C_{25}H_{30}N_4O_3$	434.2302	27.623	-5.17	0.00266
3-Morpholino-4-tetrahydro-1H-pyrrol-1-ylcyclobut-3-ene-1,2-dione	$C_{12}H_{16}N_2O_3$	236.1198	27.606	-5.36	0.01375
3,14-Dihydroxy-19-oxobufa-4,20,22-trienolide	$C_{24}H_{30}O_5$	398.2088	30.735	-5.19	0.01140
Desonide	$C_{24}H_{32}O_6$	416.2192	30.717	-4.99	0.00892
4-(Tricyclo[5.2.1.0~2,6~]dec-4-en-8-yl)phenol	$C_{16}H_{18}O$	226.1355	27.605	-5.29	0.01139
N-{[(2R,4S,5S)-5-{[4-(2-Pyrimidinyl)-1-piperazinyl]methyl}-1-azabicyclo[2.2.2]oct-2-yl]methyl}benzamide	$C_{24}H_{32}N_6O$	458.2272	31.498	-2.38	0.00657
pro-phe-arg	$C_{20}H_{30}N_6O_4$	418.2347	36.047	-9.48	2.1251E-05
4-Ethyl-2-(2-methoxyphenyl)-5-methyl-6-[3-oxo-3-(1-pyrrolidinyl)propyl]pyrazolo[1,5-a]pyrimidin-7(4H)-one	$C_{23}H_{28}N_4O_3$	408.2151	16.723	-3.14	0.00118
ethyl 1-[4-({[4-(tert-butyl)anilino]carbonyl}amino)phenyl]-3,5-dimethyl-1H-pyrazole-4-carboxylate	$C_{25}H_{30}N_4O_3$	434.2305	30.726	-4.93	0.00744
ethyl 1-[4-({[4-(tert-butyl)anilino]carbonyl}amino)phenyl]-3,5-dimethyl-1H-pyrazole-4-carboxylate	$C_{25}H_{30}N_4O_3$	434.2299	30.274	-8.94	7.7522E-07
(4beta,12R)-8-Oxo-12,13-epoxytrichothec-9-en-4-yl (2Z)-2-butenoate	$C_{19}H_{24}O_5$	332.1618	27.605	-5.4	0.01294
5,11-Diethyl-5,6,11,12-tetrahydro-2,8-chrysenediol	$C_{22}H_{24}O_2$	320.1775	28.655	-6.06	0.00195
3,14-Dihydroxy-19-oxobufa-4,20,22-trienolide	$C_{24}H_{30}O_5$	398.2088	28.463	-9.07	1.0552E-05
2-(1-Oxo-4-phenyl-2(1H)-phthalazinyl)-N-(2,2,6,6-tetramethyl-4-piperidinyl)acetamide	$C_{25}H_{30}N_4O_2$	418.2348	31.51	-2.14	0.01618
